# Real‐world first‐line systemic therapy patterns in metastatic castration‐resistant prostate cancer

**DOI:** 10.1002/bco2.129

**Published:** 2021-12-14

**Authors:** Angelyn Anton, Sruti Pillai, Marie Christine Semira, Shirley Wong, Julia Shapiro, Andrew Weickhardt, Arun Azad, Edmond M. Kwan, Lavinia Spain, Ashray Gunjur, Javier Torres, Phillip Parente, Francis Parnis, Jeffrey Goh, Olivia Baenziger, Peter Gibbs, Ben Tran

**Affiliations:** ^1^ Division of Personalised Medicine Walter and Eliza Hall Institute Melbourne Victoria Australia; ^2^ Department of Medical Oncology Eastern Health Melbourne Victoria Australia; ^3^ Department of Medical Oncology Monash Health Melbourne Victoria Australia; ^4^ Department of Medical Oncology Olivia Newton‐John Cancer and Wellness and Research Centre Melbourne Victoria Australia; ^5^ Department of Medical Oncology Western Health Melbourne Victoria Australia; ^6^ Alfred Health Melbourne Victoria Australia; ^7^ Department of Medical Oncology Peter MacCallum Cancer Centre Melbourne Victoria Australia; ^8^ Faculty of Medicine, Nursing and Health Sciences Monash University Melbourne Victoria Australia; ^9^ Department of Medical Oncology Goulburn Valley Health Shepparton Victoria Australia; ^10^ Department of Medical Oncology Adelaide Cancer Centre Adelaide South Australia Australia; ^11^ Faculty of Health and Medical Sciences University of Adelaide Adelaide South Australia Australia; ^12^ Department of Medical Oncology Royal Brisbane and Women's Hospital Brisbane Queensland Australia

**Keywords:** abiraterone, docetaxel, enzalutamide, metastatic castration‐resistant prostate cancer, real‐world data, systemic therapy

## Abstract

**Introduction:**

Several systemic therapies have demonstrated a survival advantage in metastatic castration resistant prostate cancer (mCRPC). Access to these medications varies significantly worldwide. In Australia until recently, patients must have received docetaxel first, unless unsuitable for chemotherapy, despite no evidence suggesting superiority over androgen receptor signalling inhibitors (ARSIs). Our study investigated real‐world systemic treatment patterns in Australian patients with mCRPC.

**Methods:**

The electronic CRPC Australian Database (ePAD) was interrogated to identify mCRPC patients. Clinicopathological features, treatment and outcome data, stratified by first‐line systemic therapies, were extracted. Comparisons between groups utilised Kruskal–Wallis tests and Chi‐Square analyses. Time‐to‐event data were calculated using Kaplan–Meier methods and groups compared using log‐rank tests. Factors influencing overall survival (OS) and time to treatment failure (TTF) were analysed through Cox proportional hazards regression models.

**Results:**

We identified 578 patients who received first‐line systemic therapy for mCRPC. Enzalutamide (ENZ) was most commonly prescribed (*n* = 240, 41%), followed by docetaxel (DOC, *n* = 164, 28%) and abiraterone (AA, *n* = 100, 17%). Patients receiving ENZ or AA were older (79, 78.5 years respectively) compared with DOC (71 years, *p* = 0.001) and less likely to have ECOG performance status 0 (45%, 44%, 59% in ENZ, AA and DOC groups respectively *p* < 0.0001). Median TTF was significantly higher in those receiving ENZ (12.4 months) and AA (11.9 months) compared to DOC (8.3 months, *p* < 0.001). PSA50 response rates and OS were not statistically different. Time to developing CRPC > 12 months was independently associated with longer TTF (HR 0.67, *p* < 0.001) and OS (HR 0.49, *p* = 0.002).

**Conclusion:**

In our real‐world population, ENZ and AA were common first‐line systemic therapy choices, particularly among older patients and those with poorer performance status. Patients receiving ENZ and AA demonstrated superior TTF compared to DOC, while OS was not statistically different. Our findings highlight the important role of ARSIs, given the variability of access worldwide.

## 1 INTRODUCTION

Prostate cancer is the most common non‐cutaneous cancer in males globally, associated with over one million new cases and 350,000 deaths each year.^1^ Over the past decade, the treatment landscape in castration‐resistant prostate cancer (mCRPC) has dramatically evolved following the development and approval of several life‐prolonging systemic therapies. However, access to these treatments varies worldwide.^2^, ^3^ Docetaxel (DOC), abiraterone acetate (AA) and enzalutamide (ENZ) have each demonstrated survival benefits in the first‐line mCRPC setting.^4^, ^5^, ^6^ However, these agents have not been directly compared in prospective randomised studies, and therefore the optimal treatment and sequencing strategy remains unclear. The emerging evidence for earlier use of systemic therapies, in the hormone‐sensitive setting, has also influenced subsequent treatment selection and sequences in mCRPC.

International guidelines support the choice of any approved first‐line systemic therapy, given the absence of validated predictive biomarkers.^7^, ^8^ In Australia however, the reimbursement of androgen receptor signaling inhibitors (ARSIs) was until recently, limited to patients previously treated with docetaxel, or those who are deemed unsuitable for chemotherapy. Our study aimed to examine real‐world patterns of care among Australian patients with mCRPC, focusing on first‐line treatment choices, toxicity and outcomes.

## 2 METHODS

The electronic CRPC Australian Database (ePAD) was interrogated to identify patients who received systemic therapy for mCRPC. The ePAD multi‐site clinical registry prospectively collects data including clinicopathologic details, treatment choices, response, toxicity and long‐term outcomes for consecutive patients with CRPC. Nineteen Australian healthcare sites including private and public settings in both metropolitan and regional locations were included in the registry at the time of data extraction. Data are entered into the password‐protected database by treating clinicians or trained data abstractors, following review of all relevant medical records. Data updates are performed at regular intervals.

Castration‐resistance is defined by Prostate Cancer Working Group 3 (PCWG3) criteria; progression despite androgen deprivation therapy (ADT), as defined by a rise in serum prostate‐specific antigen (PSA), radiological progression of pre‐existing disease and/or the appearance of new metastases, unless otherwise assessed by the treating clinician. First‐line systemic therapy is defined as the first additional systemic therapy received following progression on ADT and/or first‐generation anti‐androgens such as nilutamide, bicalutamide or flutamide. ‘Watchful waiting’ was defined by a period of observation without commencement of systemic therapy for at least 3 months or longer following the development of CRPC. Treatment decisions including selection and sequencing were determined by the treating clinician.

Baseline patient and disease characteristics, treatment response and outcome data were extracted from ePAD. Descriptive statistics were used to report baseline clinicopathological factors and PSA50 response rates; defined as ≥50% decline in PSA level compared to baseline, following initiation of treatment, and adverse events (AEs), stratified by treatment choice. Differences between groups were determined using Kruskal‐Wallis tests and Chi‐square analyses. Time to treatment failure (TTF) and overall survival (OS) were calculated using the Kaplan–Meier method and comparisons were made using log‐rank tests. TTF was defined as the time period from the initiation of first‐line systemic treatment until cessation of therapy for progressive disease or death. In cases where treatment was stopped for reasons other than disease progression or death, TTF was defined as the time to commencement of second‐line systemic therapy. Treatment duration was defined as the time period from the initiation of first‐line systemic treatment until cessation for any reason. OS was defined as the period from the initiation of first‐line treatment, to death from any cause.

Cox proportional hazard regression modelling was performed to analyse the effect of individual variables on TTF and OS. Variables with a *p* value of <0.1 on univariate analysis were included in the multivariate model. A *p* value of <0.01 was considered statistically significant. Prism software (version 8.3.1, GraphPad Software LLC, La Jolla California, USA) was utilised for all analyses except Cox proportional hazard regression models, which were performed using Stata/SE software (version 16.1, StataCorp LLC, Texas, USA).

## 3 RESULTS

**FIGURE 1:**
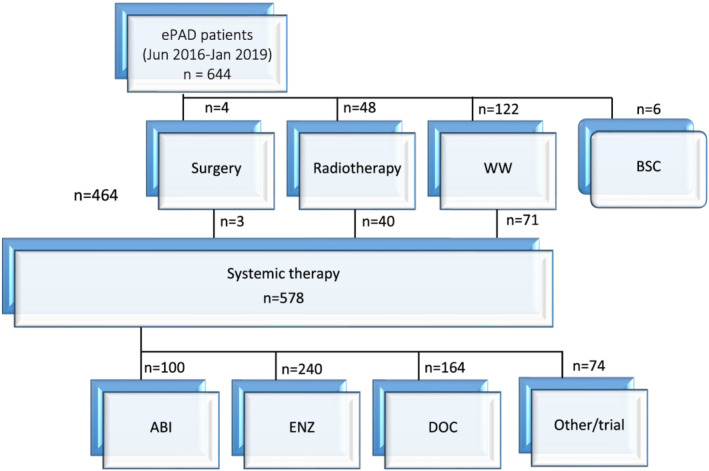
Consort diagram—initial treatment decisions following castration‐resistance

We identified 578 patients who had commenced systemic therapy for mCRPC and were enrolled into the ePAD database between July 2016 and December 2019. Median age at CRPC was 75 years (range 38–99 years) and median duration of follow up was 17.2 months. De novo metastatic disease was detected in 228 (39%) patients and visceral disease was present in 49 (8%) patients. Following mCRPC diagnosis, 464 (80%) patients directly commenced systemic treatment (Figure 1). A further 71 (12%) underwent prior ‘watchful waiting’ for ≥ 3 months, 40 (7%) underwent palliative radiotherapy and 3 (1%) had surgery prior to commencing systemic therapy.

The most common first‐line systemic therapy prescribed was ENZ (*N* = 240, 41%), followed by DOC (*n* = 164, 28%) and AA (*n* = 100, 17%). A smaller number (*n* = 74, 13%) received first‐line systemic therapy as part of a clinical trial or other systemic therapies such as carboplatin‐based regimens. Table 1 displays baseline characteristics for each treatment group, based on first‐line therapy choice.

**TABLE 1:** Baseline and other characteristics by first‐line therapy choice

	ABI	ENZ	DOC	Trial/Other	Total	*p* value
	*N* = 100	*N* = 240	*N* = 164	*N* = 74	*N* = 578	
Median Age (years)	78.5	79	71	71	75	**0.001**
ECOG Performance Status						
0	44 (44%)	108 (45%)	96 (59%)	53 (72%)	301 (52%)	**<0.001**
1	37 (37%)	103 (43%)	65 (40%)	6 (8%)	211 (37%)	
≥2	18 (18%)	28 (12%)	3 (2%)	1 (1%)	50 (9%)	
Unknown	1 (1%)	1 (<1%)	0 (0%)	14 (19%)	16 (3%)	
Comorbidities						
Hypertension	60 (60%)	137 (57%)	76 (46%)	32 (43%)	305 (53%)	0.026
Ischaemic heart disease	25 (25%)	83 (35%)	30 (18%)	16 (22%)	148 (26%)	**0.002**
Previous Stroke	16 (16%)	17 (7%)	10 (6%)	2 (4%)	45 (8%)	**0.005**
Gleason Score						
≤7	13 (13%)	61 (25%)	43 (26%)	19 (26%)	136 (24%)	0.20
≥8	42 (42%)	96 (40%)	92 (56%)	35 (47%)	265 (46%)	
Unknown	45 (45%)	83 (35%)	29 (18%)	20 (27%)	177 (31%)	
De Novo Metastatic Disease	51 (51%)	83 (35%)	69 (42%)	25 (34%)	228 (39%)	0.024
Visceral metastases	7 (7%)	15 (6%)	19 (12%)	8 (11%)	49 (8%)	0.22
Median PSADT						
<1 month	22 (22%)	49 (20%)	21 (13%)	19 (26%)	111 (19%)	0.55
1–3 months	37 (37%)	82 (34%)	62 (38%)	21 (28%)	202 (35%)	
4–6 months	18 (18%)	38 (16%)	22 (13%)	13 (18%)	91 (16%)	
>6 months	8 (8%)	19 (8%)	8 (5%)	5 (11%)	40 (7%)	
Unknown	15 (15%)	52 (22%)	51 (31%)	16 (22%)	134 (23%)	
Median PSA at CRPC Diagnosis	16.2	15.85	15.0	5.0	10.6	**0.001**
Time to CRPC (months)	46.7	64.9	47.5	29.4	48.7	**0.001**
Prior ‘Watchful Waiting’	14 (14%)	30 (13%)	18 (11%)	9 (12%)	71 (12%)	0.91
Prior Upfront Docetaxel	19 (19%)	37 (15%)	4 (2%)	13 (18%)	73 (13%)	**<0.001**
Prior Treatment						
Surgery	0 (0%)	2 (1%)	0 (0)	1 (1%)	3 (1%)	0.42
Radiotherapy	7 (7%)	15(6%)	16 (10)	10 (14%)	40 (7%)	0.20
Median Treatment Duration (months)	11.7	11.9	5.6	8.8	N/A	**<0.001**
AE During Therapy	11 (11%)	57 (24%)	43 (26%)	11 (15%)	122 (21%)	**0.01**
Median follow‐up (months)	13.8	13.1	26.2	16.8	17.2	N/A

### 3.1 Patient characteristics

Patients who received DOC or trial/other therapies were significantly younger (median age 71 years) compared to those treated with ENZ (79 years) and AA (78.5 years; *p* = 0.001) and were more likely to have an ECOG performance status of 0 (72% vs. 59% vs. 45% vs. 44% in trial/other, DOC, ENZ and AA groups respectively, *p* < 0.0001). Ischaemic heart disease history was more prevalent in the ENZ group (35%) compared with AA (25%), DOC (18%) and trial/other (22%) groups (*p* = 0.0021). Those treated with AA were more likely to have a previous history of stroke (16% vs 7%, 6%, 4% in ENZ, DOC and trial/other groups respectively; *p* = 0.005).

### 3.2 Disease characteristics

There were no significant differences between treatment groups with regards to Gleason score, PSA doubling time (PSADT) or the presence of visceral metastases. Overall, 73 patients (13%) had received upfront docetaxel in the hormone sensitive setting. Only 4 (2%) in the DOC group received prior upfront chemotherapy compared to those treated with AA, ENZ and trial/other treatments (19%, 15%, 11% respectively; *p* < 0.001). Median pretreatment PSA was lowest in the trial/other group (5.0 ng/ml) compared to the AA, ENZ or DOC groups (16.2, 15.85 and 15.0 ng/ml; *p* = 0.001). The proportion of patients who had undergone prior ‘watchful waiting’, surgery or radiotherapy before initiation of systemic treatment was similar between groups.

### 3.3 Treatment outcomes

**FIGURE 2:**
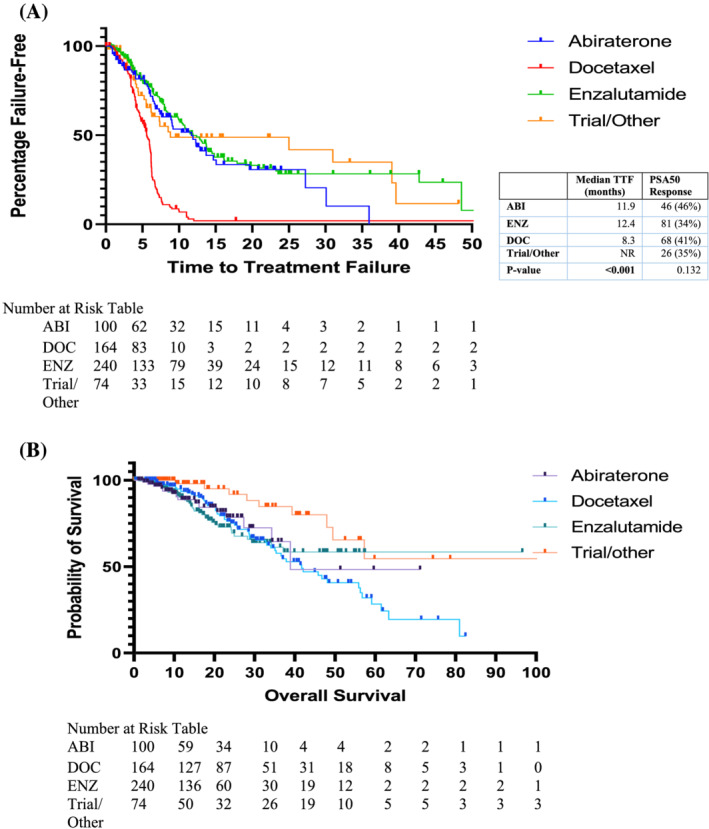
Time to treatment failure and overall survival by first‐line therapy choice

Patients treated with DOC had lower median treatment duration (5.6 months vs. 11.9 months vs. 11.7 months) compared to ENZ and AA respectively (*p* < 0.001; Table 1). Median TTF was also lower in the DOC group (8.3 months vs. 12.4 months (ENZ) vs. 11.9 months (AA); *p* < 0.001; Figure 2A). However, there were no significant differences in PSA50 response rates (41% vs. 34% vs. 46% *p* = 0.132). Patients were more likely to develop AEs resulting in dose modification, delay or hospitalisation if receiving DOC (26%) or ENZ (24%) compared to AA (11%) or trial/other treatments (15%); *p* = 0.01. Overall survival was not significantly different between groups (*p* = 0.77; Figure 2B).

### 3.4 Multivariate analyses

Univariate and multivariate analyses were performed to assess the effect of individual variables on TTF and OS (Table 2). On univariate analysis, longer time to development of CRPC > 12 months was associated with longer TTF (HR 0.67, 95% CI 0.51–0.88; *p* < 0.001). AA, ENZ and trial/other therapies were associated with significantly superior TTF compared to DOC, and this was maintained on multivariate analysis for AA (HR 0.55, 95% CI 0.35–0.86; *p* = 0.008), ENZ (HR 0.50, 95% CI 0.36–0.70; *p* < 0.001) and trial/other groups (HR 0.52, 95% CI 0.32–0.85; *p* = 0.008). The only other independent prognostic factor associated with improved TTF on multivariate analysis was time to development of CRPC >12 months (HR 0.60, 95% CI 0.43–0.83; *p* = 0.002).

**TABLE 2:** Univariate and Multivariate Analyses for TTF and OS

Variable	Univariate HR (95% CI)	*P* value	Multivariate HR (95% CI)	*P* value
TTF				
Age^a^	0.99 (0.98–1.00)	0.179		
ECOG at CRPC (ref < 1)				
≥1	1.48 (0.88–2.50)	0.14		
Ischaemic Heart Disease	0.98 (0.75–1.27)	0.86		
Stroke	0.96 (0.64–1.46)	0.862		
Hypertension	0.97 (0.77–1.24)	0.83		
Gleason Score (Ref ≤ 7)				
8–10	1.39 (1.03–1.88)	0.031	1.22 (0.90–1.66)	0.203
Time to CRPC (Ref ≤ 12 mo)				
>12 months	0.67 (0.51–0.88)	**0.004**	0.60 (0.43–0.83)	**0.002**
PSA at CRPC (Ref < 14.3)	1.11 (0.87–1.42)	0.409		
≥median 14.3 ng/mL				
PSA Doubling Time (Ref ≤ 3)	0.78 (0.59–1.04)	0.095		
>3 months				
De Novo Metastatic Disease	1.04 (0.82–1.32)	0.724		
Visceral Metastases	1.57 (1.05–2.31)	0.025	1.03 (0.65–1.65)	0.887
Prior Upfront Docetaxel	1.20 (0.85–1.71)	0.303		
First‐line Systemic Therapy				
(Ref Docetaxel)	0.46 (0.35–0.61)	**<0.001**	0.50 (0.36–0.70)	**<0.001**
Enzalutamide	0.54 (0.38–0.77)	**<0.001**	0.55 (0.35–0.86)	**0.008**
Abiraterone	0.52 (0.34–0.80)	**0.003**	0.52 (0.32–0.85)	**0.008**
Trial/0ther				
OS				
Age^a^	1.02 (1.00–1.04)	0.037	1.01 (0.99–1.04)	0.274
ECOG at CRPC (ref < 1)				
≥1	3.62 (1.88–6.97)	**<0.001**	2.56 (0.99–6.12)	0.052
Ischaemic Heart Disease	1.69 (1.15–2.50)	**0.008**	1.62 (1.04–2.51)	0.032
Stroke	1.00 (0.50–1.97)	0.994		
Hypertension	1.14 (0.78–1.66)	0.50		
Gleason Score (Ref ≤ 7)				
8–10	1.17 (0.75–1.84)	0.490		
Time to CRPC (Ref ≤ 12mo)	0.48 (0.32–0.71)	**<0.001**	0.49 (0.31–0.77)	**0.002**
>12 mo				
PSA at CRPC (Ref < 14.3)	1.52 (1.4–2.24)	0.032	1.27 (0.84–1.91)	0.252
≥median 14.3 ng/mL				
PSA Doubling Time (Ref ≤ 3)	0.75 (0.48–1.17)	0.202		
>3 mo				
De Novo Metastatic Disease	1.55 (1.08–2.23)	0.017	1.38 (0.90–2.12)	0.143
Visceral Metastases	1.76 (0.99–3.15)	0.055	1.63 (0.90–3.00)	0.113
Prior Upfront Docetaxel	1.59 (0.84–3.00)	0.153		
First‐Line Systemic Therapy				
(Ref Docetaxel)	0.93 (0.62–1.41)	0.747	0.95 (0.59–1.55)	0.852
Enzalutamide	0.83 (0.47–1.47)	0.515	0.77 (0.39–1.51)	0.449
Abiraterone	0.36 (0.18–0.73)	**0.004**	0.55 (0.52–1.18)	0.123
Trial/Other				

^a^ Continuous variable; *Significant p values < 0.01 are shown in bold*.

On univariate analysis, factors associated with inferior OS included ECOG performance status ≥ 1 (HR 3.62, 95% CI 1.88–6.97, *p* < 0.001) and history of ischaemic heart disease (HR 1.69, 95% CI 1.15–2.50, *p* = 0.008). Longer time to CRPC > 12 months was a favorable prognostic variable associated with OS on univariate (HR 0.48, 95% CI 0.32–0.71; *p* < 0.001) and multivariate analyses (HR 0.49, 95% CI 0.31–0.77; *p* = 0.002). Individual first‐line systemic treatment choices were not significantly associated with OS on multivariate modeling.

## 4 DISCUSSION

This retrospective analysis demonstrates that ARSIs, particularly ENZ are commonly prescribed as first‐line therapy in a real‐world Australian setting. This is despite the recent reimbursement restrictions, indicating that a significant proportion of real‐world prostate cancer patients are not suitable for chemotherapy. This is consistent with the differences in baseline characteristics between treatment groups within our cohort, as those receiving ENZ and AA were older and more likely to have poorer performance status or comorbidities. To our knowledge, this is the largest series reporting real‐world treatment patterns in the Australian mCRPC population. The use of ARSIs have progressively increased over the past decade and correlates with the earlier introduction of systemic therapy in mCRPC within Australia.^9^ Data from clinical registries in Europe and the United States also suggest that their use exceeds that of chemotherapy in the first‐line setting for mCRPC, although AA was prescribed more frequently than ENZ in those series.^10^, ^11^, ^12^, ^13^ However, access to these medications remains variable worldwide.^2^, ^3^

The variability of cost and access to life‐prolonging medications is an important global issue, leading to a significantly higher mortality rate in low development index countries due to the disparity in resources, including the availability of life‐prolonging therapies.^1^ In Australia, ARSIs and DOC are available treatments, however, until recently, the reimbursement of ARSIs had been restricted in the first‐line setting, influencing treatment choice. Given the significant proportion of patients receiving ARSIs in the Australian real‐world setting, the longer TTF and higher AE rates with DOC compared to AA, further cost‐effectiveness analyses are warranted to further explore the true treatment‐ and toxicity‐related healthcare costs.

To date, there have been no prospective randomised studies informing the optimal initial therapy for mCRPC and subsequent treatment sequencing decisions. While some retrospective studies have suggested that initial treatment with docetaxel chemotherapy may be superior to ARSIs with higher cancer‐specific survival,^14^ others have demonstrated superior outcomes with ARSIs compared to DOC.^12^, ^15^ For example, Chowdhury et al. recently reported a longer median time to progression with AA (9.6 months) and ENZ (10.3 months) compared to DOC (7.6 months, *p* < 0.0001) in a large multi‐centre European retrospective registry study. However, there was no difference in OS between first‐line therapy groups in this and another American retrospective study.^12^, ^13^ These results are similar to those of our study, where TTF was shorter with chemotherapy, but there were no differences in PSA50 response rates or OS.

The current mCRPC treatment landscape is limited by a lack of reliable biomarkers to guide treatment decisions. In our cohort, time to CRPC >12 months was a favorable predictive and prognostic marker on multivariate analyses for TTF and OS, in keeping with previous literature. Other documented prognostic factors in CRPC, include PSADT, Gleason score and the presence of visceral metastases.^16^, ^17^ However, these did not appear to influence the choice of systemic therapy and were not independently associated with either TTF or OS in our cohort.

When comparing our results with pivotal trials, median TTF with AA (11.9 months) and ENZ (12.4 months) was lower than the median radiographic progression‐free survival (PFS) reported in the COU‐AA‐302 (16.5 months) and PREVAIL (20 months) studies.^4^, ^18^ PSA50 response rates were also lower in our cohort, likely reflecting differences between the trial and real‐world populations. In our Australian cohort, this may reflect the limited reimbursement of ARSIs to patients deemed unsuitable for chemotherapy at the time of analysis. Similarly, recently reported real‐world data relating to ENZ demonstrated lower PSA50 response rates in chemotherapy‐naïve patients compared to the PREVAIL study.^19^ Furthermore, another European real‐world study demonstrated a lower PFS in those receiving AA of 10.8 months, likely related to the older age, higher rates of comorbidities and visceral metastases within their cohort compared to the COU‐AA‐302 study.^20^

In patients receiving DOC however, the median TTF in our cohort (8.3 months) was higher than the median PFS in the FIRSTANA study (5.3 months), with similar PSA50 response rates than the pivotal TAX 327 study.^6^, ^21^ Importantly, the median age in our cohort was slightly higher than in the prior trials, which also involved narrow eligibility criteria, excluding patients with abnormal cardiac function or significant medical conditions. This highlights the importance of recognising differences between real‐world patients compared with clinical trial populations and the challenges in extrapolating results in the real‐world setting. In fact, in our cohort, patients who were enrolled in clinical trials were younger, had fewer comorbidities and better performance status. It is also likely that they commenced treatment earlier in their disease trajectory, reflected by a lower median pre‐treatment PSA.

The higher use of ENZ compared to AA in our study contradicts observations from previous international registries.^12^, ^13^, ^14^ This likely reflects the differences in access or cost between ARSIs in many countries. For example, within several European countries the cost of abiraterone to patients is lower than that of enzalutamide due to differences in reimbursement regulations.^2^ Whereas in Australia these drugs are both reimbursed equally. A phase II randomised trial comparing AA and ENZ in the first‐line setting demonstrated higher PSA30 response rates with ENZ (82 vs. 68%) but no differences in time to PSA progression or overall survival.^22^ Furthermore, inferior patient‐reported outcomes relating to quality of life, fatigue and cognition have been reported with ENZ.^23^ Our study similarly demonstrated lower rates of significant adverse events with AA compared to ENZ and treatment choice reflected the known toxicity profiles. Patients receiving ENZ were more likely to have a history of ischaemic heart disease and more patients treated with AA had prior stroke history.

Subsequent therapies and potential cross‐resistance are also significant considerations when making initial treatment choices. For example, ARSIs have demonstrated survival benefits in chemotherapy‐naïve and post‐docetaxel settings. However, the use of a second ARSI following progression is associated with modest response rates.^24^ Importantly, there are conflicting data regarding the effect of prior ARSIs on docetaxel efficacy with some studies demonstrating inferior PSA50 response rates,^25^ while others have demonstrated no significant difference.^26^ The high use of ARSIs in our cohort will allow the comparison of different treatment sequences and potential cross‐resistance with longer follow‐up.

The increasing utilisation of ARSIs and/or docetaxel in the hormone sensitive setting will certainly influence time to CRPC and subsequent therapy choice and efficacy in the near future. For example, DOC re‐challenge in mCRPC following upfront chemohormonal therapy has demonstrated only modest response rates.^27^ This is reflected in the treatment choices within our cohort with only 2% of the DOC group having received prior upfront DOC. A proportion of our cohort (13%) also received first‐line therapies other than the standard‐of‐care options of DOC, ABI or ENZ, mostly through clinical trials. Several novel combination therapies have demonstrated activity signals in the mCRPC setting including targeted therapies and PARP inhibitors.^28^, ^29^ Therefore, ongoing follow‐up will enable further evaluation of outcomes in these emerging treatment groups and the changing patterns of care in the real‐world setting.

We acknowledge the limitations of our retrospective study, including the small cohort size and short follow‐up period. Our endpoint of TTF was pragmatic, relating to clinical decision‐making where cessation of treatment or change to the next line of therapy is driven by variables surrounding the treating clinician's practice, rather than the pre‐defined endpoints of clinical trials utilising strict PSA and imaging criteria. As a result, clinicians may often continue treatment beyond minor progression if a patient continues to derive clinical benefit, especially given the favourable toxicity profile of ARSIs. Patient preference may also influence this practice, particularly considering the potential toxicities of chemotherapy and in some settings, the need to change clinicians for those who may initially be managed primarily by urologists, or the need to travel long distances to receive chemotherapy, for those living in regional or remote locations. Furthermore, there may be a lower threshold for commencing ARSIs in those treated with prior docetaxel. These factors are likely to contribute to a longer TTF in those receiving ARSIs. As such, the objective endpoint of OS was included and is also clinically relevant when comparing treatment options. Whilst cross‐trial comparisons of PFS in previous clinical trials and TTF in our study are hypothesis‐generating, potential confounding factors cannot be wholly accounted for in this retrospective design.

The ePAD registry is Australia's largest advanced prostate cancer registry and continues to recruit patients from a selection of sites representative of the wider Australian population. We believe the real‐world treatment patterns and comparative efficacy of systemic therapies is of great clinical interest. However, OS data remain immature and longer follow up is required to evaluate the influence of further systemic therapies and differences between treatment sequences. Registry data are also limited by the accuracy of medical records, where comorbidities, ECOG and toxicity data are often under‐reported. Furthermore, the database does not include additional serum markers including haemoglobin, albumin, alkaline phosphatase or lactate dehydrogenase, nor the quantification of disease volume, which are documented prognostic factors in prostate cancer.^30^ Despite this however, we have demonstrated important differences in patient characteristics between treatment groups and when compared to clinical trial populations.

Overall, this retrospective real‐world study is an important reflection of current clinical practice in the Australian mCRPC setting. Our findings highlight the importance of real‐world data and the need to consider individual patient and disease factors in guiding treatment decisions. It also has implications regarding the reimbursement of novel therapies worldwide. While cost is an important consideration for government approval for reimbursement of novel agents, projected costs based on modelling and the uptake of novel agents may not always reflect real world practice. Our data suggest that a significant proportion of real‐world patients are not suitable for chemotherapy and therefore patients in countries without access to life‐prolonging ARSIs remain significantly disadvantaged. Further follow up is required to evaluate the influence of subsequent therapies and treatment sequences on long‐term outcomes as well as the impact of earlier introduction of systemic therapies in the hormone‐sensitive setting.

## ETHICS STATEMENT

The ePAD project was granted multisite ethics approval by the Royal Melbourne Hospital Human Research Ethics Committee on 01/05/2015, prior to commencement of data collection. The approval included a waiver of the requirement for individual patient consent given the retrospective, non‐interventional and de‐identified nature of the research and the patient group (advanced cancer) HREC/15/MH/352.

This study was conducted in accordance with the Australian National Health and Medical Research Council's (NHMRC) National Statement on Ethical Conduct in Human Research (2007) and was carried out according to the principles of the Declaration of Helsinki.
